# “Step by Step” Toward Recovery for People with Severe Mental Illness – The Example of a Community Mental Health Structure on the Outskirts of Lisbon

**DOI:** 10.1192/j.eurpsy.2025.824

**Published:** 2025-08-26

**Authors:** M. Andrade, F. Ramalheira, A. Nunes, L. Gil

**Affiliations:** 1 Hospital Júlio de Matos - ULS São José, Lisbon, Portugal

## Abstract

**Introduction:**

Addressing severe mental illness requires assertive community intervention. The “Assertive Program – Step by Step”, implemented in 2016, involves a mental health team in the Sintra region (Lisbon). Through a case manager model and regular patient contact, an Individualized Care Plan is developed to ensure treatment continuity, coordinate psychosocial interventions, and reduce both the frequency and duration of hospital admissions. The goal is to contribute to clinical improvement, enhance social functioning, and improve the quality of life for individuals with severe mental illness through several measures: regular psychiatric consultations; promotion of adherence to psychopharmacological treatment; individual or group psychological support; participation in rehabilitation programs; psychoeducational programs for patients and families; and facilitation of general medical consultations and social support.

**Objectives:**

This study aims to characterize the sociodemographic profile, occupational status, and number of hospital readmissions among patients followed by the Assertive Program, and to reflect on the relevance of these interventions in preventing relapses.

**Methods:**

A retrospective analysis of data collected from the clinical records of patients enrolled in the Assertive Program in September 2024, with a minimum follow-up period of one year.

**Results:**

In September 2024, a total of 29 patients were enrolled in the Assertive Program, with 19 receiving follow-up for more than one year. The average age was 36.4 years, and 68.4% were male. The majority of patients were either single (68.4%) or divorced (21.1%), and most were not working, with 52.6% being unemployed and 5.3% retired. The predominant diagnosis was schizophrenia (52.6%), followed by Bipolar Affective Disorder (31.6%) and Psychosis Not Otherwise Specified (10.5%). The average number of total hospital admissions was 2.9 (maximum 12, minimum 0). After joining the Assertive Program, 68.4% (n=13) of patients were not readmitted to the hospital. Of those readmitted (31.6%; n=6), most had a diagnosis of schizophrenia (n=4) and were unemployed (n=5).

**Conclusions:**

This study highlights the specific sociodemographic profile of patients with severe mental illness, who appear to be predominantly single and unemployed. The proposed program may help reduce the number of relapses in the care of this patients. Hospital readmissions appear to occur primarily among unemployed patients, underscoring the need for close, personalized follow-up, with a focus on improving occupational functionality.

**Disclosure of Interest:**

None Declared

